# Progressing Pollutant Elution from Snowpack and Evolution of its Physicochemical Properties During Melting Period—a Case Study From the Sudetes, Poland

**DOI:** 10.1007/s11270-016-2797-z

**Published:** 2016-03-17

**Authors:** Daniel Kępski, Marek Błaś, Mieczysław Sobik, Żaneta Polkowska, Katarzyna Grudzińska

**Affiliations:** Department of Polar and Marine Research, Institute of Geophysics, Polish Academy of Sciences, 64 Księcia Janusza, Warsaw, PL-01452 Poland; Department of Climatology and Atmosphere Protection, University of Wroclaw, 8 Kosiby, Wrocław, PL-54621 Poland; Department of Analytical Chemistry, Gdansk University of Technology (GUT), 11/12 G. Narutowicza, Gdansk, PL-80233 Poland; Faculty of Geoengineering, Mining and Geology, Wroclaw Institute of Technology, 15 Na Grobli, Wroclaw, PL-50421 Poland

**Keywords:** Snow cover, Water percolation, Ionic pulse, Pollutant deposition, Western Sudetes

## Abstract

Main aim of the work assumed recognition of physicochemical changes in snowpack occurring during the melting period. Properties of snow cover had been identified at two sites in Western Sudetes mountains (860 and 1228 m asl) in SW Poland since the end of January, and monitored until the disappearance of snow in late Spring. Snow pit measurements and sample collection at both sites were made followed by chemical analyses with the use of ionic chromatography. The results were compared for subsequent stages of snowpack evolution. Thermometers installed above the ground during summer in one site (860 m asl) helped to identify the thermal gradient existing inside snow during winter. During studies, special attention was paid to the pollutant elution with determination the different release rates of individual ions from the snow cover. Results of chemical analysis showed that during the thaw, the first portions of meltwater were responsible for drainage into the ground a substantial part of the impurities. During the first two weeks of thaw at higher elevated site, pollutants released from the snow cover load amounted to 123.5 mMol·m^−2^. In those days, there was a release to the ground of approximately 74, 74, and 57 %, respectively of H^+^, NO^3−^, and SO_4_^2−^ ions contained in the snow cover, while only 14 % of snow mass in the form of meltwater was released.

## Introduction

Snow cover is a specific “warehouse” of atmospheric pollutants, and, for this reason, it is especially important in higher parts of mountains, where snow may constantly persist for a few months. The flux of atmospheric pollutants to the ground via precipitation particles in a liquid and solid form is called wet deposition. Precipitation particles include, in their composition, pollutants present in clouds and also scavenge part of contamination existing below the base of clouds (Lei and Wania 2004). The existing snow cover is built, besides solid precipitation particles, of any forms of hydrometeors deposited on the surface both in a solid and liquid physical state.

In the absence of snow melting, there is a continuous accumulation of the whole contamination load in the snow cover. However, during snowmelt, the chemical composition of the water reaching the ground in a given place, is not simply the sum of the chemical components contained in consecutive portions of preceding precipitation. Very important is also the redistribution process of snow mass together with the contained impurities, which is effected by the occurring aeolian processes. Obviously this means reduction of pollutant deposition in places affected by snow erosion and increase, where wind-induced deposition occurs. Partial melting of the snow cover from the top or bottom and draining of part of this water to the ground can also occur. Additionally, on the snow surface, both dry and fog deposition of pollutants take place. All these processes lead to constant changes in the snow cover chemistry. Determining the composition of pollutants present in samples retrieved from snow pits, allows understanding of the role of most important factors that influence the snow chemistry on a particular terrain.

The release of pollutants from snow cover occurs efficiently only during the ablation season. During that time take place penetration of meltwaters with solutes into the soil, groundwater, and surface water. First portion of such contaminated water, which carry a large fraction of the soluble ions that reach the environment beneath snow is often called “ionic pulse” (Liu et al. 1997). The importance of that phenomenon is dependent on factors like the rate of the melting of the snow cover (Colbeck 1981; Bizzotto et al. 2009), the frequency and the depth of the following freezing and refreezing cycles (Tsiouris et al. 1985; Bales et al. 1993; Lee et al. 2008), the concentration of pollutants (Brimblecombe et al. 1987; Domine and Thibert 1995), the structure of the pollutants in the snow cover (Colbeck 1981; Bales et al. 1989), the water redistribution in the snow (Bales et al. 1990; Harrington et al. 1996), the temporal changes of snow metamorphism (Davis 1991; Hewitt et al. 1991), the transfer of heat energy deep into the snow cover (Williams et al. 1996), the measurement method and the sampling manner (Marsh and Pomeroy 1993). In short time of a few days, this results in removal from the snow cover of a large amount of the accumulated pollutant load and in large environmental stress (Williams et al. 2009). Intensity and resulting negative effects of such stress are dependent on geological structure and the vulnerability of specific species of plants and animals on a given place.

The main purpose of this study is to present ongoing changes of physicochemical properties taking place in melting snowpack together with its influence on the rate and size of pollutant deposition coming from snow cover. In this regard evolution of the snow cover in Western Sudetes was examined in several snow pits in two sites during the winter season 2011/2012. This winter had clearly defined phases of the snow cover development, which made it favorable for such investigations. An important task was also determining the removal rate of the atmospheric pollutants accumulated in the snowpack, with special consideration given to the first phase of the ablation season. It was also possible to determine the release rate of individual ions from the melting snow cover. Chemical analysis results from this study were compared with data from different works localized around Northern hemisphere (Barbaris and Betterton 1996; Nickus et al. 1998; Kuhn 2001; Kang et al. 2004; Williams et al. 2009). Changes in physicochemical properties in snow profiles were related to meteorological conditions obtained from nearby meteorological sites and from installed on one snow pit place instruments. This allowed to precise identification of periods that were especially important for the snowpack evolution and for pollutant elution. Occurrence of significant thaws during winter made this task easier and gave an opportunity to compare changes in snow cover evolution in neighboring places, from which one was affected by “normal” aerial percolation stopped by thick ice layer inside snowpack, and second was the percolation columns area, where water soaked through internal channel rapidly to the ground. To our knowledge, the results presented here show for the first time clearly visible distinction, between evolution of snow inside and outside percolation columns area together with different elution rate of pollutants from these places.

## Materials and Methods

### Study Area

**Fig. 1:**
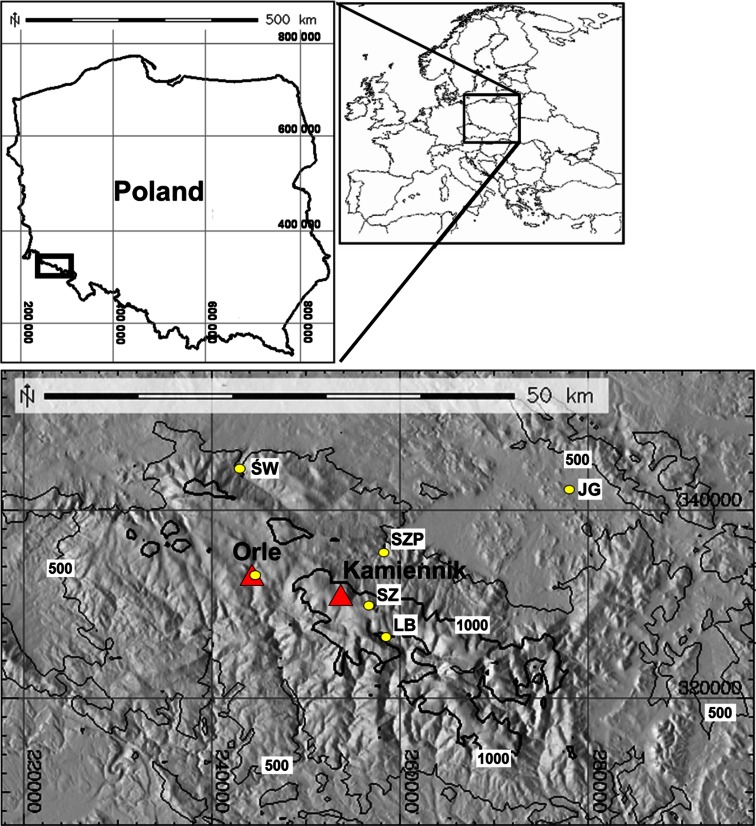
Distribution of measurement points: the physical and chemical descriptions of the snow are marked as *red triangles*, meteorological sites in the IMGW and CHMU measurement networks are marked as *yellow dots*

Meteorological conditions during the winter season 2011/2012 were characterized on the basis of the multiannual average 1961–1990 based on observation data from the Meteorological Observatory of Wroclaw University at Szrenica in the Karkonosze Mountains (Piasecki 1995). To analyze the spatial distribution of the snow cover depth, measurements from the six meteorological sites belonging to the Polish IMGW (5) and Czech CHMU (1) measurement networks were used (Fig. 1).

For the purposes of the realized research topic, two measurement sites in the terrain (Fig. 1) were organized, which functioned during the whole winter season 2011/2012. The first was Orle (OR) settlement in the Jizera Mountains (860 m asl)—a small, flat basin located in the SE part of the Polish Jizera Mountains—the meteorological conditions monitored were:

- Daily measurement of the precipitation sum (Hellmann rain gauge);
- Daily measurement of the snow cover depth;
- Automatic registration of meteorological parameters with the use of a Campbell station (temperature, air humidity, wind speed, and direction, shortwave and longwave radiation balance);
- Occasional snow pit measurements made on 31/01, 01/03, 15/03, 27/03, and 17/04. Pits from March were doubled and performed also within found there percolation columns.

**Fig. 2:**
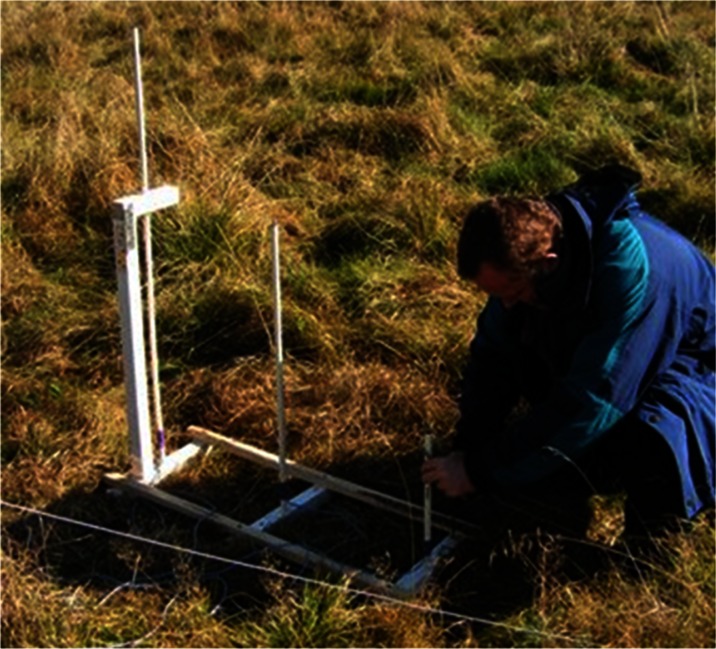
System for measuring the snow temperatures—40, 80, and 120 cm above ground

The sensors measured weather conditions at a height of approximately 2 m above the snow cover. Air temperature and humidity (HMP35, Vaisala, Helsinki, SF), a LI 200 SA shortwave radiation sensor (Li-Cor Inc.), a NRLite net radiation sensor (Kipp & Zonen, Delft, NL), as well as horizontal wind speed (Vector Instruments, Alfreton, UK) sensors were mounted. Snow temperature (PT100 probe) was registered at three depths (40, 80, and 120 cm above the ground, Fig. 2). All data were recorded from 10 min sampling intervals using a CR10X data logger.

The second was Kamiennik (KA, 1228 m asl)—a site on a convex landform situated c.a. 30 m below Kamiennik peak in Karkonosze on a slope angled 12° with NE aspect. It was the fixed place for snow pit digging, which were made on 1/02, 29/02, 15/03, 27/03, 17/04, and 8/05.

### Methodology

#### Physical Properties Measurements in Snow Pits

A detailed description of the physical and chemical properties of the snow in a vertical profile was performed according to The International Classification for Seasonal Snow on the Ground (Colbeck et al. 1990). During the snow pit investigations, the size and shape of snow crystals and their density, hardness, temperature, and liquid water content were determined. For density measurements was used 10 cm height snow density cutter and scaled analog Newton meter. Each separate layer was measured individually. Temperature was obtained from needle temperature probe for each 10 cm starting from surface to the ground. Liquid water content and hardness was estimated from hand test in accordance with the current measurement methods (Colbeck et al. 1990; Fierz et al. 2009). Moreover, the collected material formed the basis for the calculations of snow water equivalents (from density) and the total load of pollutants (from samples collected for each separate layer). Dates of measurements were chosen to catch important stages of the snow cover evolution, e.g., stabilization of the snowpack depth after prolonged accumulation period or the beginning of an intense ablation season.

#### Water Drainage Detection with Thermal Imaging Camera

For visualization of the water drainage in the snow cover, a thermal imaging camera, T420 bx of the FLIR Company, which operates in the infrared spectrum, was used. Detectors of thermal imaging cameras work like a matrix of individual detectors (called pixels). Each of these detectors converts radiation falling on it to a measurement signal from which the surface temperature of the emission source is determined. Thermal images (thermogram) present the distribution of the snow temperature on the exposed surface of the snow pit wall. For the quality of the thermogram result, the so-called emissivity factor is decisive. Knowing this quantity allowed obtaining temperature values with the accuracy guaranteed by the manufacturer.

Thermal scanning was performer at OR site during fieldworks in March, using an infrared camera (FLIR, 620 series; FLIR SystemsCo. Ltd., St Leonards, NSW, Australia) with a resolution of 640×480 pixels and accuracy of 2 °C. The camera was mounted on a tripod, and thermal imaging was performed from a fixed distance of approximately 1 m from the snow pit face. Before each thermal scanning session, the emissivity value was set to 0.98, and thermograph resolution was calibrated to ambient temperature and humidity as per manufacturer’s recommendation. The software allowed the user to obtain temperature at a specific area on the image, and calculated the minimum, maximum, and average temperatures, and standard deviation for each measuring field. The software calculated the standard deviation of each image within a defined shape by considering each pixel within the given shape (Talukder et al. 2014).

#### Analytical Procedures—Ionic Chromatography

The analysis included a number of chemical elements, i.e., pH, electric conductivity (σ), and the concentration of some major anions (F^−^, Cl^−^, NO_2_^−^, NO_3_^−^, SO_4_^2−^, PO_4_^3−^) and cations (Na^+^, K^+^, Mg^2+^, Ca^2+^, NH_4_^+^). The selected anions and cations were quantified against synthetic rain standard using ion suppressed chromatography (ICS 3000, Dionex Corporation, USA). The synthetic standards are Reference Material No. 409 (BCR-409, Institute for Reference Materials and Measurements, Belgium) and Analytical Reference Material Rain (National Water Research Institute Environment, Canada) (Polkowska et al. 2005). The detailed analytical procedure and the problems connected with it are described in Namieśnik et al. (2007). Calculations of the balance of charges were made in order to check whether an important element of the chemical composition of dew was not omitted in the conducted analysis. The PDI parameter (Percentage Difference of Ionic Balance) is one of the tools used for this aim (Cini et al. 2002). Literature data indicated that a PDI value not exceeding 20 % is the criterion of acceptability (Zimmermann et al. 2006). Taking all measurement sites during the analyzed period into account, this parameter reached a maximum value of 7 %. The obtained result confirms the high quality of the conducted analyses. Values of pH was measured in laboratory after snow samples melting with usage of microcomputer pHmeter CI-316 produced by ELMETRON company.

### Meteorological Background

#### General Characteristics of the Winter Season in Western Sudetes

The share of solid precipitation out of the annual precipitation sum in Western Sudetes is clearly connected with the altitude above sea level and amounts to 27 % at the base of the Karkonosze mountains (Jelenia Góra) and above 70 % at the top of Śnieżka (Kwiatkowski 1985). In the upper parts of the massif, due to high precipitation sums in the winter months accompanied by negative temperatures, snow cover remains unmelted for a relatively long time and has a large thickness and density. The average number of the days with snow cover during the year, according to Kosiba (1949), is 94 at the height of 600 m asl, quickly increasing to 170 at the height of 1100 m asl and finally reaching 176 days on the plateau (1500 m asl). The highest water equivalent in the snow occurs a few days after the maximum measured depth and is in a range between 80 mm at the height of 600 m asl and above 800 mm at the height of 1200 m asl. In the concave terrain forms of the upper parts of the slopes (nival niches, cirques) and along the upper limit of the forest, the snow water equivalent is much higher. It can amount to as much as 1574 mm, as in 1976 in Szrenica Cirque (Głowicki 1977) quite close to the designated in this study KA site. In the study area a large humidity deficiency is often observed in conditions of anticyclonic weather and the air subsidence in the middle and lower troposphere. Therefore, ablation takes place mainly by sublimation, which consumes almost an order of magnitude more energy than the melting. In the final result, the reduction of the water equivalent in such situations is small, and this process does not lead to a loss of the included chemical components in the snow.

The period with the persistence of snow cover in Western Sudetes can be divided into characteristic phases: initial growth (lasting on average to the second week of December), stabilization (generally to the middle of February), second growth with annual maximum (till the middle of March) and ablation (basing on Piasecki 1995).

#### Characteristics of the Winter Season 2011/2012 in Western Sudetes

**Fig. 3:**
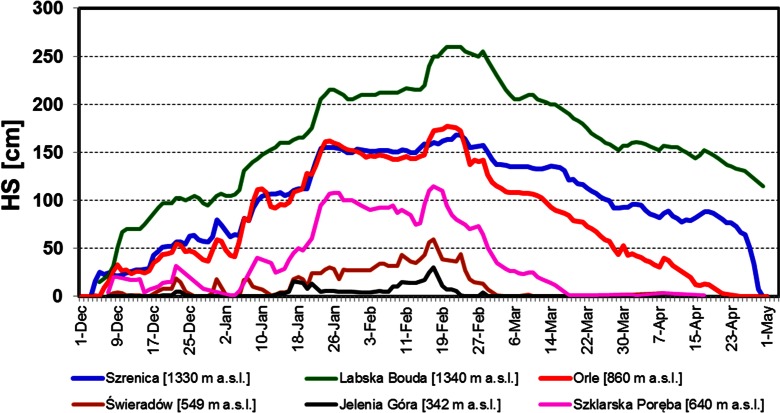
Variations of snow cover thickness in Western Sudetes during the 2011/12 winter season

**Fig. 4:**
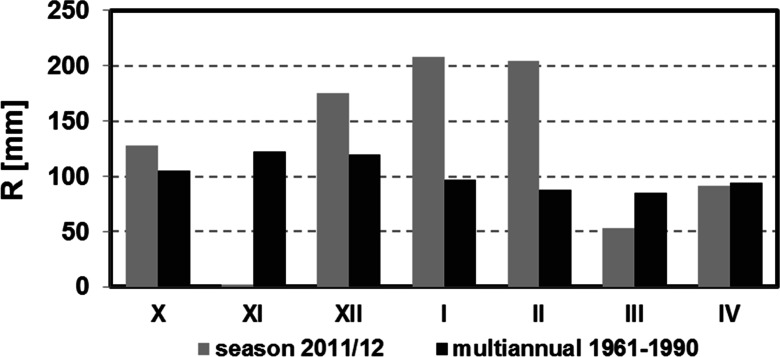
Precipitation sum on Szrenica in the winter season 2011/2012 with a comparison to the long-term average from years 1961–1990

The development of snow cover in Western Sudetes during winter 2011/2012 was substantially different from the average conditions. Except for the station at the top of Śnieżka Mountain, autumn episodes of snow settled on the ground were not observed (Fig. 3). This was caused by the extremely low precipitation sum in November 2011 that amounted to only 1 mm in middle and lower parts of the mountains and not even 10 mm on Śnieżka (Fig. 4). A prolonged period of drought ended in first days of December when the dominating anticyclonic weather pattern changed rapidly to a western cyclonic circulation pattern with an inflow of Polar maritime air masses and led to the onset of permanent snow cover in the middle and higher parts of Western Sudetes (Fig. 3). Conditions remained the same also in January with the exception of the last five days of the month (Fig. 4).At the beginning of third week of January, the height of the snow cover amounted to around 160 cm in Orle (OR) and Szrenica and 120 cm at the top of Śnieżka (Fig. 3).

In the period from 26 January to 15 February 2012, the polar maritime air from the west was replaced by continental air masses from the east. At that time, for the whole Western Sudetes area, very cold weather reigned with almost no precipitation. At the beginning of the second half of February, western atmospheric circulation with quite intense snowfalls, changing later into rainfalls, returned. In the last days of the month, warming with rainfalls was reported even in the highest parts of the mountains. In such conditions, between the 16 and 22 February 2012 for almost all stations, the seasonal maximum depth of snow cover occurred. It amounted to 30 cm in Jelenia Góra, 177 cm in Orle, 168 cm in Szrenica, and 152 cm at the top of Śnieżka (Fig. 3).

After the last week of February a steady decline in the height of the snow cover was observed. The period with continuous snow cover ended on 21 February in Jelenia Góra, the 23 April in Orle, 30 April in Szrenica, and 2 May at the top of Śnieżka. Especially intensive ablation occurred during a few days in late April and early May when an inflow of hot air from southern and eastern Europe took place (Fig. 3).

Due to the climatological conditions of winter 2011/12, December, January, and February were warmer than usual in the middle and especially the lower parts of the mountains in Western Sudetes. In the highest part, thermal conditions did not deviate substantially from the norm. Precipitation, except for in the lower parts, was significantly higher than usual, amounting 177 mm in Jelenia Góra and 535 mm at the top of Śnieżka.

## Results

### Changes of Snow Cover Physical Properties During the Winter Season 2011/12

**Fig. 5:**
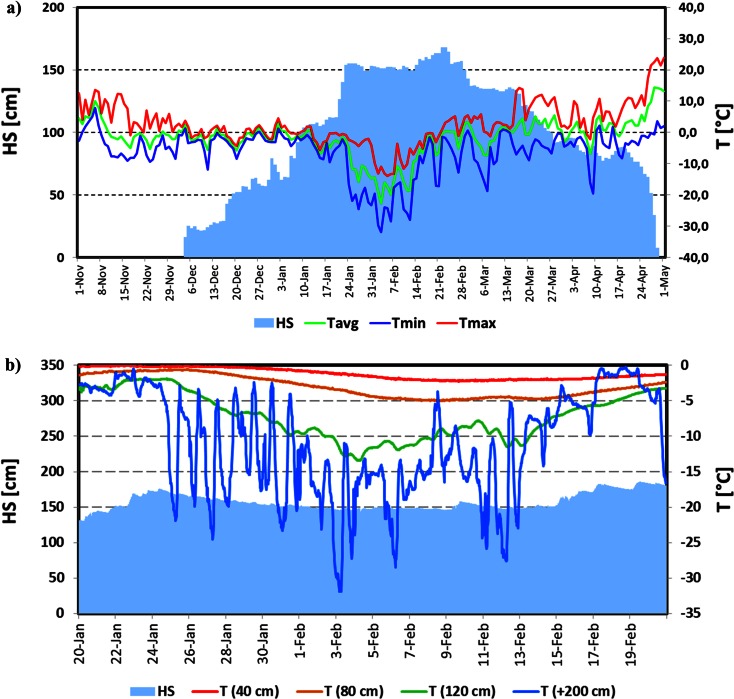
Variations of the diurnal values of average (*T*_avg_), minimum (*T*_min_), maximum (*T*_max_) temperature, and snow cover depth (Hs) in the winter season 2011/2012 (**a**); variations of the snow temperature measured 40, 80, and 120 cm above the ground, air temperature 200 cm above the snow surface between 20 January and 20 February (**b**)

**Fig. 6:**
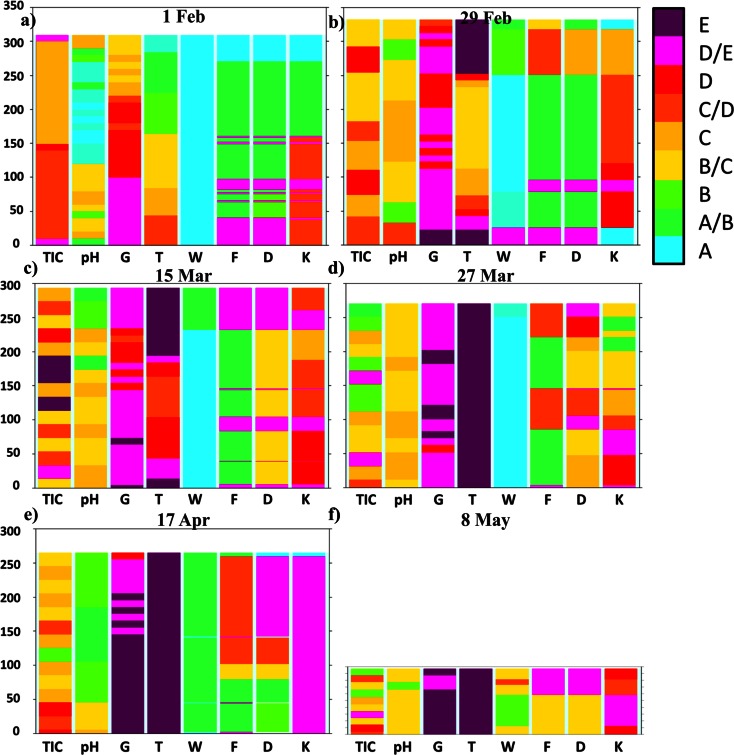
Physicochemical properties of the snow in the vertical profile at the KA site. See also Table 1 for the legend explanation

**Fig. 7:**
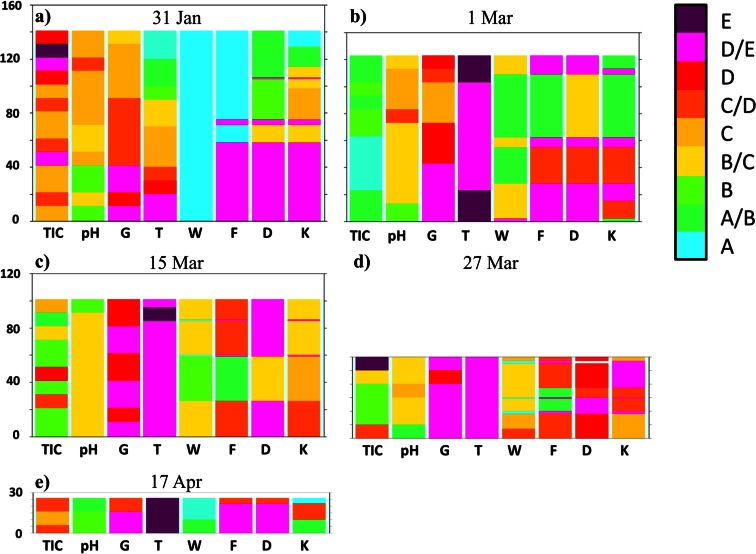
Physicochemical properties of the snow in the vertical profile at the OR site. See also Table 1 for the legend explanation

Continuous snow cover in the winter season 2011/2012 in Western Sudetes settled late—in the first days of December. However, the intense precipitation which occurred in December and January caused a fast growth of the snow depth (Figs. 3 and 5). During this period thaws were relatively short (2–3 days) and not very effective. Therefore, wet metamorphism did not play an important role in changing the physical properties of the snow cover, especially at higher elevated areas like the Kamiennik (KA) site (Fig. 6 and Table 1). During measurements made on 1 February in a 309 cm deep snow pit, dry, fine, and medium grained crystals were found in the snowpack. The high density of snow was caused by the saltation, defragmentation, and finally compaction of snowflakes produced by strong wind action. Short thaws left a mark in the snowpack in the form of a few fine ice crusts in the lower parts of the snow cover visible as thin belts with an “E” or “D/E” category running through columns F, D, and K in the bottom part of the profile (Fig. 6a). At the OR site, located 400 m lower, the mentioned thaws were more pronounced, resulting in the presence of a thicker, 40 cm deep, metamorphosed snow layer at the base of the profile (Fig. 7a).

**Table 1:** Explanation of the graphs presenting the physicochemical properties of the snow cover

	TIC [μMol dm^−3^]	pH	G [g dm^−3^]	T [°C]	W [%]	D [mm]	K [N]
A	<20.0	<3.00	<0.075	<−20.0	0 %	<0.2	<20
A/B	20…30	3.0…3.5	0.075…0.100	−20.0…−10.0	0 %/3 %	<0.2/0.2–0.5	<20/20–150
B	30…40	3.5…4.0	0.100…0.150	−10.0…−6.0	3 %	0.2–0.5	20–150
B/C	40…55	4.0…4.5	0.150…0.200	−6.0…−4.0	3 %/3–8 %	0.2–0.5/0.5–1.0	20–150/150–500
C	55…70	4.5…5.0	0.200…0.250	−4.0…−2.0	3–8 %	0.5–1.0	150–500
C/D	70…85	5.0…6.0	0.250…0.300	−2.0…−1.0	3–8 %/8–15 %	0.5–1.0/1.0–2.0	150–500/500–1000
D	85…105	6.0…6.5	0.300…0.350	−1.0…−0.5	8–15 %	1.0–2.0	500–1000
D/E	105…130	6.5…7.0	0.350…0.400	−0.5…−0.2	8–15 %/>15 %	1.0–2.0/2.0–5.0	>1000
E	>130.0	>7.00	>0.400	>−0.2	>15 %	2.0–5.0	Ice

The F parameter in the charts represents the shape of the crystals and the level of their metamorphosis (from an unchanged snow flake to a poly-crystal). (A) precipitation particles and temperature lower than 0°C; (B) fresh snow in 0°C, defragmented crystals, or situations with development of depth hoar; (C) wet metamorphism (fine and medium grains) (D) wet metamorphism (coarse grains); and (E) wet metamorphism (very coarse grains)

*TIC* total inorganic ionic content, *G* density of the snow, *T* temperature, *W* humidity, *D* grain size, *K* hardness of snow cover

After the rapid increase of the snow cover depth, a period of stabilization began. From the 25 January to the 14 February, considerable cooling, associated with anticyclonic weather and the advection of cold continental Polar and Arctic air masses, was noted. During this period, the daily minimum temperature at the OR site regularly dropped below −20 °C (Fig. 5a). Consequently, a substantial drop in the snow temperature in a large part of the profile was noted (Fig. 5a). The observed heat loss, resulting in snowpack temperature decrease, was possible due to relatively high snow density, which enhanced the thermal transmittance of snowpack. At the KA and OR sites, snow pits were made on 1 February and 31 January respectively. The snow cover depth amounted to 309 cm at KA and 141 cm at OR (Figs. 6a and 7a). At both sites, the temperature in the upper 30 cm of the snowpack dropped below −10 °C. At the same time, in the KA site, the 2 m deep layer regularly remained below -2 °C. A clear decrease of snow-cover height, amounting to 20–30 cm in the middle parts of the mountains (OR, Fig. 5b), was observed in this period. This was caused not by melting, but by the subsidence and compaction of snow. This effect was barely visible on the plateau of the mountains (Szrenica and Śnieżka, Fig. 3), where, in windy conditions, snowflakes were crushed quickly into very small particles and even the fresh snow cover had a relatively high density, which made further compaction difficult.

**Fig. 8:**
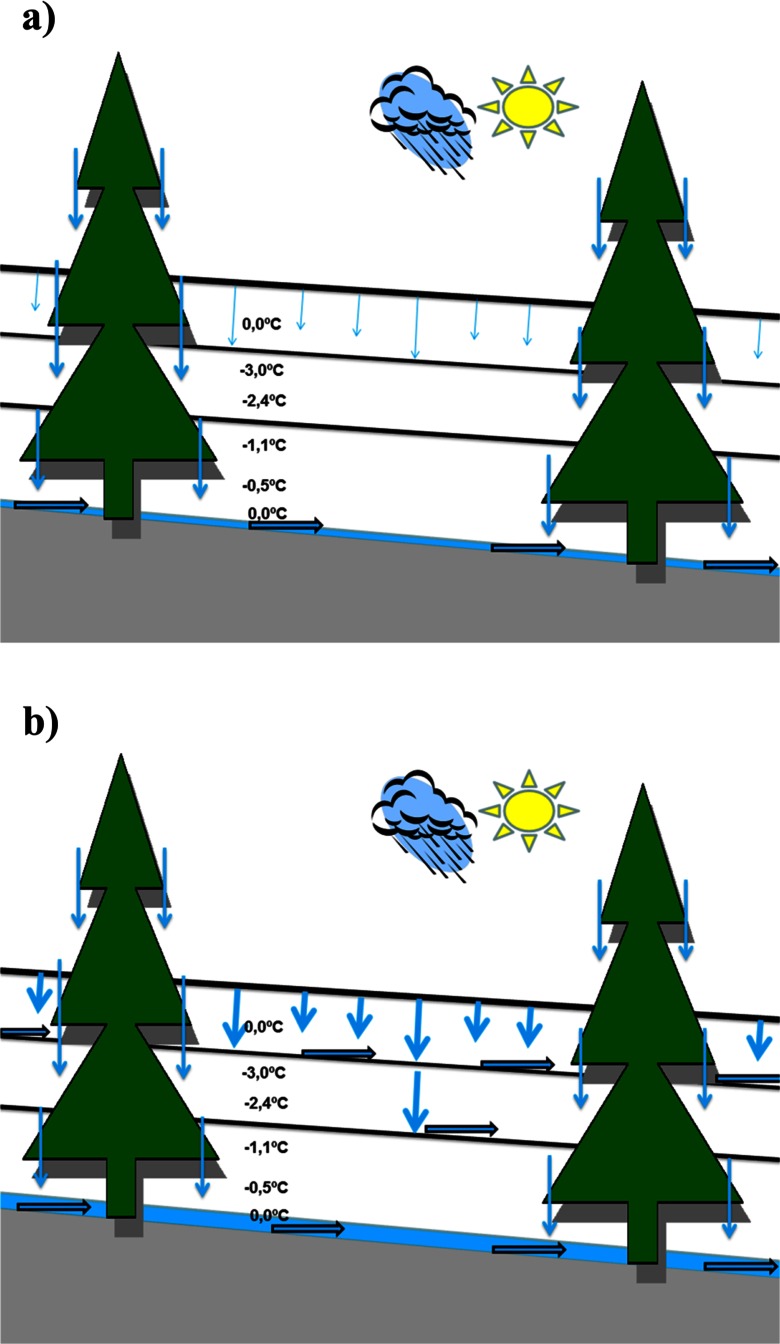
Scheme showing water redistribution in the snow cover, obtained from measurements performed in the KA and OR sites in the winter season 2011/2012: **a** first day of the thaw, start-up of a percolation column below a spruce canopy; aerial percolation limited by an ice layer; the role of the under canopy precipitation with a large share of horizontal precipitation and **b** second day of thaw—drainage of water taking into account the role of ice layers

A radical change in the development of the snow cover occurred in the last days of February, when an intense thaw began and was accompanied by large volume of rainfall—the precipitation sum amounted to 30 mm. Between 28 February and 3 March, the maximum temperature in Orle exceeded 5 °C every day (Fig. 5a). To identify the changes in the physical properties of the snow, snow pits were made at both sites—on 29 February at KA and 1 March at OR. In both cases, in upper parts of the profile (percolation zone) a wet and strongly metamorphosed coarse-grained layer of snow was formed (Figs. 6b and 7b). At the KA site, the layer being under the influence of wet metamorphism had depth of nearly 60 cm, but, at the OR, it was only 15 cm on March 1, and around 40 cm on the 15th. Therefore, an upper layer of snow with the presence of percolation was documented. The part below this layer was not touched by a “wetting front” and remained without significant changes in its internal structure. This indicates that an ice layer was formed through the contact of soaking water with the still cold snow in the base of the aerial percolation layer, which very effectively limited further percolation to the ground, but also led to the internal downhill drainage of water on the cold layer surface (Fig. 8). Snow temperature measurements at the KA site showed that, while in the surface layer of wet snow (approximately 60 cm) the temperature was 0 °C, deeper layers (60 to 170 cm below the surface) predominantly showed temperatures in the range from -3.0 °C to −2.0 °C. At the OR site internal structure of snow temperature was similar but the cold layer below the percolation zone stayed within −0.5 to −0.2 °C range.

**Fig. 9:**
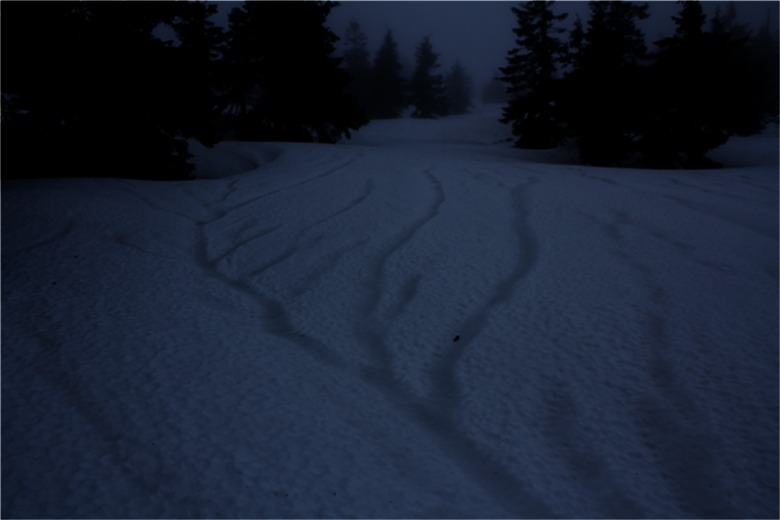
Water drainage system visible on the surface of the snow cover—36 h after the beginning of the thaw; a day with intense rainfall (∼30 mm); neighborhood of the KA site (29 Feb)

**Fig. 10:**
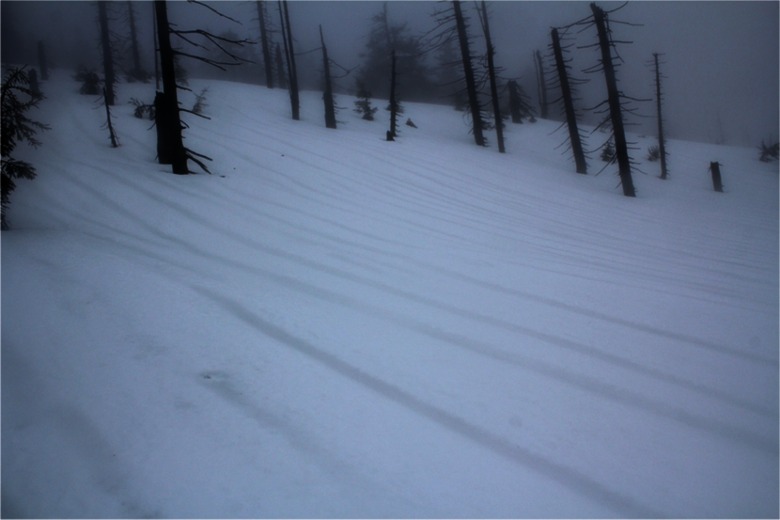
Parallel water drainage lines visible on the surface of snow cover—36 h after the beginning of the thaw; a day with intense rainfall (∼30 mm); neighborhood of the KA site (29 Feb)

During the fieldwork between 29 February and 1 March, our attention was caught by easily visible, slightly darker stripes on the snow surface (Figs. 9 and 10). Additional snow pits were dug out in such places and revealed that they were lines of much more efficient drainage of water coming from both rainfall and aerial melting within the surface layer of the snow cover. Frequently these drainage lines ended, when they met a tree protruding through the thick snow cover. Observations conducted in the terrain, allowed documenting the occurrence of isolated percolation columns below the crowns of individual spruce trees. This was a result of breaking the layers’ continuity in the snow cover, by a given tree trunk, and the occurrence of more efficient liquid precipitation under the crown of a given tree, consisting of rainfall, as well as horizontal precipitation, and melting of snow deposited in the tree canopy. It should be stressed that the horizontal precipitation from fog and clouds is especially important close to the upper limit of the forest (Błaś and Sobik 2005). A schematic diagram of the described phenomena is presented in Fig. 8.

**Fig. 11:**
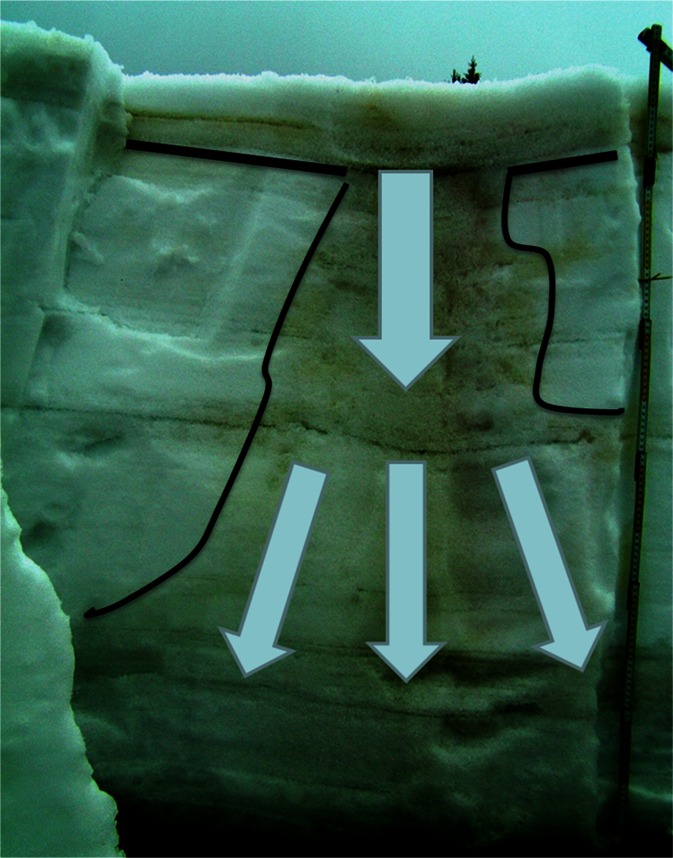
A percolation column visible in the central part of an exposed snow pit wall at OR site (2.5 days after the beginning of the thaw, a day with intense rainfall; approximately 30 mm of precipitation). For better visualization, the surface layer of snow was dyed with potassium permanganate (1 Mar)

Consecutive snow pits documented a very slow rate of aerial percolation inside the deeper layers of snow cover and important role of water and pollutant drainage by permanently functioning percolation columns close to tree trunks or, which is less common, just below the surface drainage lines. The latter were observed only on a meadow without trees at the OR site with a much gentler slope than the KA site. With the assistance of a snow dyeing method, it turned out, that percolation columns allowing the penetration of water directly to the soil were present in some places just below such drainage lines. This means that percolation at grassy areas such as OR, which usually goes down evenly, this time—in winter with exceptionally cold snow cover—was limited to water drainage lines. These were the only places where percolation reached the ground and resulted in a real deposition of pollutants (Fig. 11).

Despite the fast progressing thaw in the second half of March and in April, it was still visible that the deeper snow layer was not actually involved in wet metamorphism processes. On charts presenting the physicochemical properties of snow cover, this is visible in the middle and bottom part of profile (Figs. 6c–e and 7c, d; fine and medium grained snow marked with green and yellow colors in columns F and D).

**Fig. 12:**
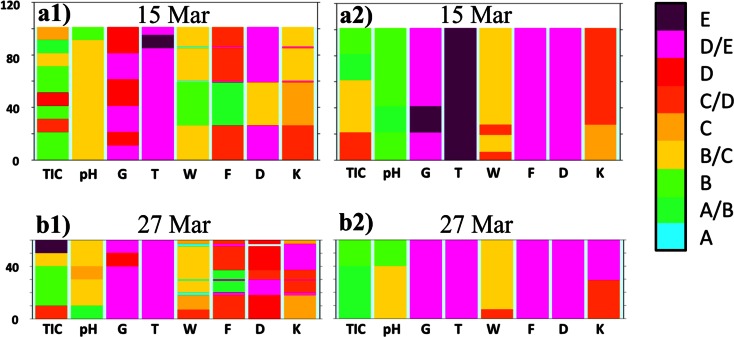
Physicochemical properties of the snow in the vertical profile at the OR site: **a** 15 Mar and **b** 27 Mar; Number *1*—snow pit dug in an area without any percolation column, number *2*—a snow pit dug in a percolation column area; explanations for the legend contained in Table 1

**Fig. 13:**
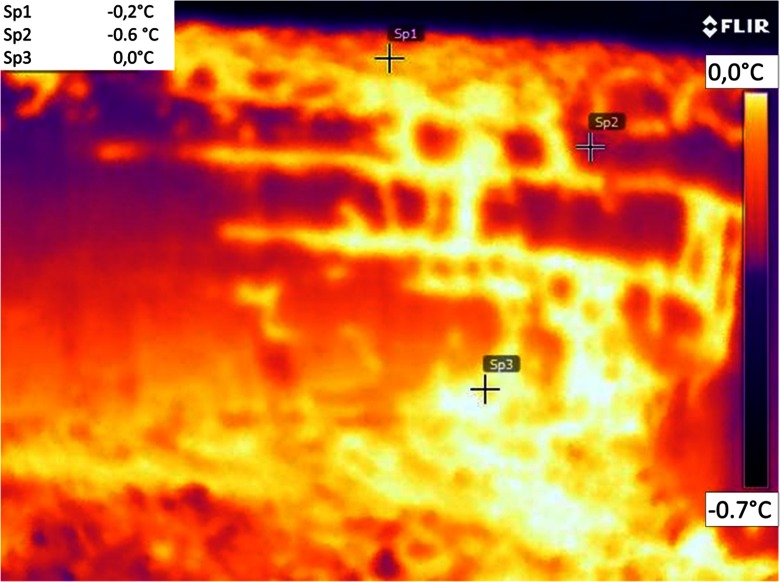
Distribution of the snow temperature on the surface of a snow pit wall, created by the use of a thermal camera at the OR site on 1 Mar

In Fig. 9, the physicochemical characteristics of snow at Orle are compared between two neighboring sites in a distance of a few meters: outside a percolation column and inside the column. Measurements showed significant differences among these parts of the snow cover, where aerial percolation was limited by the existing ice layers, and those parts with percolation columns (Fig. 12). In the first case, a layer of weakly metamorphosed snow in the middle part of the profile is clearly visible (green and yellow colors in columns F and D). In the second case, highly metamorphosed snow is present within the whole profile of the percolation column, consisted of coarse and very coarse grains in the form of polycrystals. A stoppage of percolation on the ice layer was visible in photo taken by a thermal camera (Fig. 13). In the upper part of the profile, a soaked layer of snow with a temperature equal to 0 °C is perfectly visible. However, levels with a temperature around −0.5 °C, are noticeable below. An ice layer was formed based on the contact between these two media, which later limited the vertical range of percolation. Only in places where the ice layer was not continuous, infiltration of the water inside the profile in the form of percolation columns did occur (middle and bottom part of the thermogram).

### The Role of Snow Cover in the Deposition of Atmospheric Pollutants in the Winter Season 2011/12

**Fig. 14:**
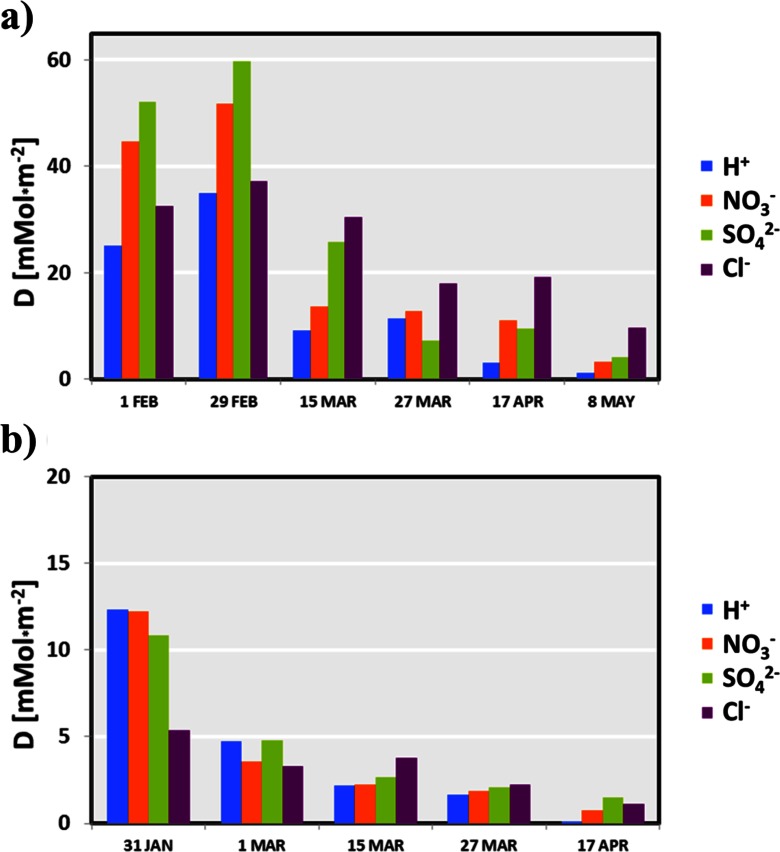
Calculated load of the selected pollutants stored in the snow cover in the winter season 2011/2012; KA site (**a**) and OR site (**b**)

Release of pollutants from snow cover and their penetration into the soil and surface water occur only during snow melting, thus until then pollution deposited in snow can be gradually accumulated through following precipitation episodes as well as dry deposition. In the Karkonosze Mts., due to the longtime of formation and high amount of stored water, snow cover has a great influence on the water flux and deposition of pollutants. To show seasonal evolution of snowpack chemistry, the total load of several major ions are presented during following snow pit measurements at KA and OR sites (Fig. 14) completed by more hydrochemical variables and water equivalent in the existing snow cover (Table 2).

**Table 2:** Snow cover water equivalent (SWE), pH, electrolytic conductivity (EC), and contamination load included in snow cover in winter season 2011/2012

	SWE	TIC	pH	EC	Contamination load									
					Cl^−^	NO_3_^−^	SO_4_^2−^	Na^+^	NH_4_^+^	K^+^	Mg^2+^	Ca^2+^	H^+^	Ʃ
	[mm]	[μMol l^−1^]		[μS cm^−1^]	[mMol m^−2^]									
	KA site													
1 February	1087	222	5.98	13.4	32.5	44.6	52.1	36.1	23.4	0.7	3.9	22.6	25.1	241.0
29 February	1454	190	5.11	10.5	37.2	51.8	59.8	33.3	17.4	1.8	4.6	34.9	34.9	275.7
15 March	1249	122	5.28	8.2	30.5	13.6	25.7	16.9	15.5	4.6	11.8	24.5	9.1	152.2
27 March	1267	73	5.12	8.1	18.0	12.8	7.2	12.9	13.5	0.9	8.2	8.5	11.4	93.4
17 April	1324	64	5.78	6.0	19.1	10.9	9.4	7.5	10.3	1.5	12.5	10.9	3.0	85.1
8 May	512	78	5.39	3.3	9.7	3.3	4.0	2.6	7.5	0.5	5.0	6.2	1.1	39.9
	OR site													
31 January	467	142	5.06	8.5	5.4	12.2	10.8	6.3	3.1	0.1	1.7	8.6	12.3	66.5
1 March	465	71	5.14	6.8	3.3	3.6	4.8	4.4	1.9	0.3	1.6	2.6	4.7	33.0
15 March	404	83	5.31	4.8	3.8	2.2	2.7	2.8	4.8	0.7	1.9	5.4	2.2	33.6
27 March	262	80	5.31	5.6	2.2	1.9	2.1	2.3	3.9	0.2	2.4	3.9	1.6	20.9
17 April	99	101	5.92	9.9	1.1	0.7	1.5	0.7	3.3	0.1	0.1	1.3	0.1	10.0

The highest ionic charge in the winter season 2011/2012 was measured on 29 February at the KA site, and it amounted to as much as 275.7 mMol m^−2^ for the sum of the analyzed ions (Table 2). Such a huge value derives from high snowpack depth (309 cm) and the water equivalent, which reached 1454 mm. The biggest share in ionic structure was sulfates and nitrates (59.8 and 51.8 mMol m^−2^, respectively). Next were ions, whose origin should be associated with sea aerosols: Cl^−^ and Na^+^ (37.2 and 33.3 mMol m^−2^). Such a large share is a consequence of dynamic cyclonic circulation and the advection of the Polar maritime air mass, which resulted in very efficient snowfalls. Next in ionic structure were Ca^+^ and H^+^ cations with a charge equal to 34.9 mMol m^−2^.

It is worth to note that one month earlier (1 Feb) the ionic charge was only slightly lower (241 mMol m^−2^) and snow water equivalent (SWE) was significantly smaller (1087 mm), which means that the concentration of pollutants on 1 Feb was highest reaching 222 μMol l^−1^ for the sum of analyzed ions and only 190 μMol l^−1^ on 29 Feb. This can be explained by the fact, that there was no intense melting before 1 Feb and the total pollution load in snow gradually increased. The snow pit of 29 February was made during ongoing melting, which caused partial outflow of water with chemical components to the ground. This resulted in the decrease of pollutant concentration. During following snow pit measurements until the final melting of the snow cover in May, the observed drop of chemical load was faster than the decrease of SWE.

Comparing the pollutant load stored in the snow cover before and after the thaw, it can be estimated how much contaminations were released to the soil at a particular time. The obtained results indicate that, between 29 February (intense phase of thaw) and 15 March, the greatest load of pollutants, i.e., 123.5 mMol m^−2^ (Table 2, Fig. 14) was released at the KA site. The highest load decrease was observed for NO^3−^, SO_4_^2−^, and H^+^ ions: −38.2; −34.1, and −25.8 mMol m^−2^, respectively. In case of H^+^ ions, this corresponds to approximately annual buffer capacity of the igneous rocks (Agren 1988; Nilsson 1988) from which the Karkonosze and Jizera Mts. are built (Kasiński et al. 2004).

These results mean that the share of hydrogen ions measured in the snow cover on 15 March was already 74 % lower than on 29 February. Significant changes also concerned nitrates (74 % decrease) and sulfates (57 %). Meanwhile, the loss of other ingredients was small, for example Cl^−^ load was reduced only by 18 %. Similar dependence was also found in the OR site (Table 2, Fig. 14) and in successive phases of the snow cover melting. However, at the OR site two important differences are visible: (1) both the concentrations and load of pollutants were lower than at KA site; and (2) the highest SWE, concentrations and load were measured in the first snow pit on 31 January. It can be accounted for higher air temperature at lower altitude (OR) than at KA site, which caused larger number and intensity of thaw episodes and subsequently led to earlier and more intense removal of the chemical ingredients.

## Discussions

In ecosystems experienced by increased deposition of atmospheric pollutants, it is important to recognize not only the size, but also the real rate of deposition. This becomes important in case of Karkonosze, which are built of the acidic granite rocks with low real buffering capacity. Deposition calculated on the basis of pollutant concentrations and the quantity of diurnal precipitation does not reflect its actual influence on the environment. This applies primarily to the winter season, when, due to snow retention, pollutants are accumulated. This means that their release occurs as late as during the spring thaw. This is extremely important, e.g., in mountain areas, where snow can persist for a few months and the share of solid precipitation is significant on an annual scale.

Particularly informative in this respect is the experiment performed in Western Sudetes during the winter season 2011/2012. It showed the great influence of weather variations on the development of snow cover and on the redistribution of water and the rate of contamination release. A sharp division of winter stages helped in proper designation of pollutants accumulated in snowpack and their subsequent elution. First snow pit was dug before any significant thaw at both study sites, which makes chemical measurements adequate to pollutants deposition until that time. Later weather pattern led to the occurrence in snow cover an extremely intense and unusual percolation event and the ionic pulse phenomenon connected with it. At the contact between surface layer affected by percolation and the layer of strongly supercooled snow below, an icy zone was formed. Its presence limited the possibility of aerial water percolation through the snow cover. This led to the activation of very efficiently working surface drainage system and percolation columns. Water driven by gravity flowed along the inner ice layer, forming water drainage lines, which were visible on the surface of the snow in the form of dark lines. The most effective were the isolated percolation columns existing around trees, which allowed the drainage of water and contaminations down to the ground.

Additionally, at less steep terrain without trees, some vertical percolation columns existed below drainage lines, which was possible thanks to the pressure of flowing water causing cracks. A series of performed measurements in the subsequent stages of the thaw revealed that the presence of the mentioned ice layer substantially limited changes in snow physical properties and the vertical range of wet metamorphism. Actually until the end of the winter season, the ice layer was a kind of a protective shield against wet transformation and changes of physical properties advancing deep into the snow cover. The wet metamorphism occurred only in the percolation zone above the ice layer and in the range of percolation columns. Such situation allowed for comparison of different physical properties and pollutant elution inside percolation column to the neighboring area, which have not been described before so clearly. Results presented in this work showed generally lower concentration of pollutants and acidification inside percolation columns than in neighboring area (Fig 12). However, more contaminated were bottom layers of snowpack inside the percolation columns, while outside the area of percolation columns higher concentration of pollutants was observed above the existing ice layer (Fig 12 a1, a2). Later snow pits in the same area showed further pollutants scavenging, which led them to move down through snowpack outside percolation column area and caused almost disappearance of them within mentioned column (Fig 12 b1, b2). The process of contamination transport through the snow cover is not well recognized and requires further studies with higher time resolution. Results presented here had an objective to define properties of snow cover during few stages of its seasonal development and it was achieved quite well. However, completely different results of snowpack properties below spruce canopy versus more open terrain showed that the choice of the correct spot for reliable measurements is extremely important and can change the sense of the whole work.

The rate of pollutant deposition released from snowpack is strictly depended on the number and intensity of thaws. This is because pollutants contained in snow cover, are generally not integrated with the snow crystal structure, but have tendency to stay in pores between the crystals in an aqueous solution with increased concentration. In lower temperatures, more water in the pores freezes, causing a higher concentration of the remaining aqueous solution (Obled and Rosse 1977). During the ablation of the snow cover, the amount of water in a liquid state increases. This first causes a saturation of the snowpack; subsequently, water begins to penetrate into the ground. Hence, the first water portions released from the snow cover to the soil and the ground and surface water should have a much higher ionic concentration in relation to the average concentration in the existing snowpack (Hibberd 1984).

The release of such a large pollutant load in a short time could lead to a dramatic lowering of pH in soil and surface waters. This is especially dangerous when the buffer capacity of acid substances is low. This applies to, e.g., granite, from which the Karkonosze Mountains are built. In mountain ranges built from carbonate rocks, which are rich in calcium, potassium, and magnesium, the ability to buffer acidic deposition is much higher. While carbonate rocks can buffer even above 200 mMol H^+^ on the surface of 1 m^2^ per year, in the case of igneous rocks like granite or gneiss, this capacity is estimated only at a level of approximately 20–30 mMol m^−2^ r^−1^ (Agren 1988; Nilsson 1988). For this reason, in Karkonosze, where increased acidic atmospheric deposition occurs, significant acidification of the soil was observed, covering the whole of its layer down to the bedrock (Sobik 1999). This is one of the direct causes of deforestation, as observed in Western Sudetes in the 1980s and 1990s. Similar problems can be found also in the USA (Cadle et al. 1984; Singh and Singh 2001), Canada (Jeffries et al. 1979), Norway (Skartveit and Gjessing 1979), and Sweden (Dickson 1980), where seasonal growth of acidification in rivers and lakes was observed.

Differences in individual ions release rate, resulting from the ionic pulse phenomenon, were documented also during research conducted by Davies et al. (1982), Brimblecombe et al. (1985), and Tranter et al. (1986, 1987). Singh and Singh (2001) supposed that the rate of release of subsequent ions should be related to their integration into snow crystal particles. For instance, a condensation nucleus in the form of sea salt aerosols is most often trapped inside an ice crystal, but these ingredients, which are scavenged during the falling of snowflakes through the atmospheric boundary layer (first of all SO_4_^2−^ and NO_3_^−^), are attached only to the surface of the crystal. This means that, during thawing, they are the first ones released (Tsiouris et al. 1985; Tranter et al. 1986). Singh and Singh (2001) also pay attention to fact that Na^+^ and Cl^−^ are easily soluble in ice. This also means that they are least mobile during a thaw. The confirmation of these relationships, through results obtained from ice cores collected in the central Antarctica, indicates a pronounced decrease of nitrogen in the surface layer of ice (Mayewski and Legrand 1990; Wagnon et al. 1999).

The importance of ionic pulse phenomenon was well described in the literature. The results obtained from this study showed much higher deposition of pollutants in chosen study area than in any reference place from other studies (Table 3). More probable state of the environment shows results from OR site, due to greater compatibility of snow water equivalent in snow cover with measured precipitation (Figs. 4 and 5). In KA site such high values of SWE and contamination load per square meter was a result of snow redistribution by wind, enhanced by location of this place near the upper forest limit. Acquired data still show an ongoing problem with atmospheric pollution in Western Sudetes, which led to huge deforestation in the 1980s. Such huge ionic content, even few times higher than in the Alps, apart from the significant environmental pollution caused by nearby industrial region (Worobiec et al. 2008), is probably the result of enhanced wet deposition in the study area, that is affected by frequent wet air masses resulting in the orographic fog (Błaś and Sobik 2004). Additionally, at this elevation above sea level, the clouds form more often during the winter season (Błaś et al. 2002). It was proved that deposition of pollutants is more efficient from rime and liquid fog deposit accumulation than by precipitation itself (Polkowska and Sobik 2008). This, together with the fact that Western Sudetes form the first larger barrier on the way of wet (and contaminated) air masses from the west (Sobik and Blas 2008), explains high total ionic content of winter snowpack. It is worth to mention that such large amount of pollutants was achieved during only two months of snow cover existence, where in areas like Himalayas or Alps, contamination was much lower beside the fact that pollutants could be accumulated a few months longer. Despite much larger contamination load present in studied snowpack, elution of pollutants proceeded completely ordinarily. On the basis of two snow pits examined on 29 February and 15 March at the KA site, it can be concluded that there was a release to the ground of approximately 74, 74, and 57 %, respectively, of H^+^, NO_3_^−^, and SO_4_^2−^ ions with the decrease only 14 % of SWE. It fits well with previous research findings. It was proved before, that first 10 % of meltwater may drain up to 80 % of the soluble contents out of snowpack (Liu et al. 1997). Laboratory experiment performed by Davis et al. (1995), showed removal from 69 to 85 % of H^+^, 70 to 86 % of NO_3_^−^, and from 71 to 89 % of SO_4_^2−^ ions with the first 20 % of SWE decrease. Various values were dependent on established conditions of melting. On the other hand, similar to our fieldwork studies conducted by Bales et al. (1990), showed elution from about 75 to 90 % of ions, when SWE decreased by 10 %.

**Table 3:** Chemical content of snow (μleq L^−1^) at maximum snow accumulation from KA (for 29 February, so after first weak pollutant elution) and OR site (for 31 January) compared to representative areas of the Northern Hemisphere (adapted from Williams et al. 2009 after Edwards et al. 2007)

	Contamination load								
	Cl^−^ [μleq L^−1^]	NO_3_^−^	SO_4_^2−^	Na^+^	NH_4_^+^	K^+^	Mg^2+^	Ca^2+^	H^+^
This study 2011—Sudetes KA	25.6	35.6	82.3	22.9	12.0	1.2	6.3	48.0	24.0
This study 2011—Sudetes OR	11.6	26.1	46.3	13.5	6.6	0.2	7.3	36.8	26.3
Williams et al. (2009)—2006 Colorado	1.5	10.9	6.3	2.1	3.3	0.8	5.7	35.0	1.9
Williams et al. (2009)—2007 Colorado	1.8	12.1	7.7	1.9	5.6	6.5	2.6	10.2	5.5
Nickus et al. (1998) —Alps	0.6	2.8	1.7	0.2	0.7	0.0	0.2	0.5	5.5
Kuhn (2001) —Alps	3.5	9.8	4.5	3.6	5.9	0.4	2.9	18.5	13.1
Kang et al. (2004)—Himalayas	0.7	0.2	0.5	0.0	0.0	0.0	0.1	0.1	NA
Kang et al. (2004)—China	1.7	5.7	1.6	1.7	1.7	0.4	0.4	6.1	2.9
Barbaris and Betterton (1996) —Arizona	7.9	11.8	18.1	4.6	21.2	2.2	2.3	6.3	12.0

## Conclusions

This paper shows that the weather pattern has a great impact on the rate of the pollutant release from the snow cover. During this study rapid thaw after long persistence of low temperatures led to the formation of unusually clear drainage system and significant differences in snowpack physicochemical properties between neighboring locations. Results from Western Sudetes showed much higher concentration of pollutants than in similar studies conducted in other parts of world, but release of pollutants proceeded ordinarily according to the results of previous research papers, with faster elution of sulfuric and nitrogen than chloride ions.

Snow pits performed before and after thaw event, showed elution of much more than half ionic components from snowpack. That means the first portions of water coming from the snow cover that infiltrate to the soil and the surface and ground water brought a substantial load of pollutants. This in turn implies that, during the spring thaw, the most intense acidification of soil and water ecosystems occurs. It is greater in places where the water amount stored in the snow is larger and where the concentration of pollutants is higher. The most important effect of the “ionic pulse” occurs in areas with the longest presence and the highest depth of snow cover, as in the neighborhood of the upper limit of the forest and in concave terrain forms.

In the end, it should be stated that, while snow cover development during winter and maximum height and snow water equivalent change significantly from year to year, the basic factors related to the additional acidification of the environment under the influence of snow ablation at the end of any winter are similar.
